# Long term efficacy of first-line afatinib and the clinical utility of ctDNA monitoring in patients with suspected or confirmed EGFR mutant non-small cell lung cancer who were unsuitable for chemotherapy

**DOI:** 10.1038/s41416-024-02901-6

**Published:** 2024-12-05

**Authors:** Sanjay Popat, Adam Januszewski, Mary O’Brien, Tanya Ahmad, Conrad Lewanski, Ulrike Dernedde, Petra Jankowska, Clive Mulatero, Riyaz Shah, Jonathan Hicks, Tom Geldart, Mathilda Cominos, Gill Gray, James Spicer, Karen Bell, Simon Roitt, Clive Morris, Yenting Ngai, Laura Hughes, Allan Hackshaw, William Wilson

**Affiliations:** 1 The Royal Marsden NHS Foundation Trust, London, UK; 2 Imperial College London and St Bartholomew’s Hospital NHS Trust, London, UK; 3 Imperial College London, London, UK; 4 Royal Free London NHS Foundation Trust and University College London Hospitals NHS Foundation Trust, London, UK; 5 James Paget University Hospitals NHS Foundation Trust, Great Yarmouth, UK; 6 Somerset NHS Foundation Trust, Somerset, UK; 7 Leeds Teaching Hospitals NHS Trust, Leeds, UK; 8 Maidstone and Tunbridge Wells NHS Trust, Maidstone, UK; 9 Beatson West of Scotland Cancer Centre, Glasgow, UK; 10 University Hospital Dorset NHS Foundation Trust, Poole, UK; 11 East Kent Hospitals University NHS Foundation Trust, Canterbury, UK; 12 Norfolk and Norwich University Hospitals, Norwich, UK; 13 Kings College London, London, UK; 14 Inivata Ltd, Cambridge, UK; 15 CR UK & UCL Cancer Trials Centre, London, UK

**Keywords:** Non-small-cell lung cancer, Prognostic markers

## Abstract

**Background:**

Here we present long-term outcomes of first line afatinib in comorbid patients with suspected or confirmed EGFR mutant NSCLC otherwise considered unsuitable for chemotherapy, and the clinical utility of serial ctDNA monitoring.

**Methods:**

TIMELY (NCT01415011) was a multicentre, single arm, phase II trial conducted in the UK. Patients aged ≥18 were treated with daily oral afatinib (40 mg) until disease progression or unacceptable toxicity. Blood samples for ctDNA analysis were obtained at baseline and 12-weekly until treatment discontinuation. The primary endpoint was PFS.

**Results:**

Thirty-nine patients were enrolled between March 2013 and August 2015. Median follow-up was 98 months (range 69-101). Median PFS was 7.9 months (95% CI 4.6-10.5). Seven patients (18%) continued afatinib beyond 18 months, 3 beyond 36 months and 2 were still on treatment at last follow-up 101 months post-treatment initiation. Analysis of baseline ctDNA samples identified 8 EGFR mutant cases that were not identified by tissue genotyping and ctDNA clearance was associated with improved PFS and OS.

**Conclusion:**

Afatinib is a viable treatment option for tissue or ctDNA-detected EGFR mutant NSCLC comorbid patients, with a proportion achieving long-term clinical benefit. Plasma ctDNA testing improved EGFR mutant identification and its clearance predicted improved PFS and OS.

## Background

Approximately 40% of newly diagnosed non-small cell lung cancer (NSCLC) patients in England do not receive anticancer treatment, largely due to a combination of advanced disease, restrictive performance status and comorbidities [1]. Diagnostic tumour sampling is often not possible in these unfit patients who are predominately treated with best-supportive care and have poor outcomes.

Epidermal growth factor receptor (EGFR) mutant NSCLC is a distinct biological entity that should be preferentially treated with an EGFR tyrosine kinase inhibitor (TKI) [2]. TKIs are attractive agents for treating medically unfit NSCLC patients as they have demonstrated marked clinical efficacy with a favourable safety profile [3–6]. Whilst osimertinib is the favoured EGFR TKI of preference for patients with common EGFR mutations, in many regions its cost remains prohibitive and its use is limited.

Afatinib is a second-generation TKI which has been shown to improve progression-free survival (PFS) for patients harbouring EGFR mutations compared to platinum-doublet chemotherapy in the first-line setting [7, 8], and also has efficacy against uncommon EGFR mutations [9], where it still plays a major role. Two phase II trials have assessed the effect of afatinib as first-line treatment of fit elderly patients with EGFR mutant NSCLC, with both demonstrating a PFS benefit compared to historical control [10, 11], and there are promising outcomes in real-world populations [12–14]. There are limited data however on its efficacy and tolerability among those with comorbidities; specifically, those deemed otherwise unsuitable for chemotherapy.

The identification of predictive genetic alterations is often problematic in NSCLC given the small size of diagnostic biopsies and high prevalence of cytological-based diagnoses [15]. An international survey found that 77% of newly diagnosed patients with advanced NSCLC in the UK were tested for EGFR mutations and that 21% of patients for whom a test was ordered were started on first line treatment before the results were available [16]. These data are reflected globally [17] and has become compounded by the additional number of genetic alterations needing genotyping for routine first-line decision-making in advanced NSCLC. Reasons for inadequate testing included histological subtype, insufficient/poor quality tissue and co-morbidity-associated poor performance status (PS) limiting tissue sampling.

Circulating tumour DNA (ctDNA) genotyping with increasingly sensitive and specific platforms, offers a less invasive alternative to tumour biopsies, affords more rapid turnaround times over tissue genotyping [18] and allows for serial monitoring of tumour genotype throughout treatment.

The TIMELY trial was initiated in 2013 and designed to investigate the safety and efficacy of afatinib in NSCLC patients with comorbidities considered unsuitable for chemotherapy and who had suspected or confirmed EGFR mutation. At the time, ctDNA testing did not exist, single gene EGFR tissue testing was becoming more prevalent with meaningful genotyping failure rates, erlotinib was still approved for unselected NSCLC post chemotherapy, and for patients with failed (unknown) EGFR status clinically unsuitable for chemotherapy, the only management strategy available was supportive care. Osimertinib did not exist at the time the trial was run. We focus on the long-term durable benefit of afatinib, and the clinical utility of ctDNA monitoring.

## Methods

### Study design and patients

We conducted a multicentre, single arm, open label, phase II trial. Eligible patients were aged ≥18 years and had either (i) confirmed EGFR mutation [any activating mutation between EGFR exons 18–21] and WHO Performance Status (PS) 0–3 or (ii) suspected EGFR mutation (no tissue for genotyping/failed genotyping), along with adenocarcinoma, WHO performance status (PS) 0–2, and were never/former-light smokers. Never light smoking criteria were used as per CALGB30406 to maximize underlying EGFR mutation rate [19]. Patients were locally-deemed medically unsuitable for platinum-doublet chemotherapy due to frailty or comorbidities. Reasons for chemotherapy unsuitability was not collected. Patients previously treated with an EGFR inhibitor or anti-cancer systemic therapies for advanced NSCLC, and those suitable for radical radiotherapy, were ineligible. Palliative radiotherapy was allowed.

This trial was sponsored by University College London and funded by Boehringer Ingelheim. The trial was approved by the Scotland A Research Ethics Committee (REC Reference no. 11/SS/0092) and the Medicines for Human Use Regulatory Agency, UK (EudraCT no. 2011-003608-19) and was conducted in accordance with Good Clinical Practice and the Declaration of Helsinki. Written informed consent was obtained from each patient before enrolment. The trial was registered in a public registry; ClinicalTrials.gov identifier: NCT01415011.

### Treatment and study procedures

Patients received daily oral afatinib (40 mg) in 28-day cycles until disease progression or unacceptable toxicity. Dose reductions for treatment-related toxicities were allowed (minimum dose of 20 mg). CT scans were performed 4 weeks after starting treatment and then every 8 weeks for the first year and 12-weekly thereafter until progression, or when clinically indicated. Plasma samples for ctDNA were obtained at baseline and 12-weekly until treatment discontinuation.

### Endpoints

The primary endpoint was progression-free survival (PFS) at 6 months, defined as the time from registration until disease progression or death from any cause. Patients who did not progress or die were censored at the date last seen. Secondary endpoints included overall survival (OS), best overall response according to RECIST 1.1, treatment compliance and adverse events (graded using CTCAE version 4.0). OS was measured from registration until death from any cause, otherwise censored at the date last seen. Treatment compliance was summarised by time on treatment, measured as the time from treatment initiation to cessation.

### Translational aspects

Pre-planned exploratory analyses in the diagnostic formalin-fixed paraffin embedded tissue samples and plasma samples collected at baseline, every 12 weeks during treatment and upon progression were performed retrospectively. The laboratory research was conducted in collaboration with Imperial College of Science, Technology and Medicine Molecular Genetics laboratory, London, the Institute of Cancer Research, London and Inivata Ltd, Cambridge.

We assessed the molecular aberrations in diagnostic tissue samples and investigated the role of ctDNA next generation sequencing (NGS) and monitoring using plasma. DNA was isolated from the FFPE blocks using Qiagen extraction kits. The DNA concentration was determined for quality control and if QC requirements met, sequencing libraries generated utilising the Trusight Tumour 170 targeted capture assay. The libraries were sequenced on Illumina TruSeq and sequencing results were run on an inhouse pipeline for QA and variant calling.

At each patient’s timepoint for sample collection, plasma was isolated into ~5 × 1.5 ml cryovials from ~2 x 9 ml whole blood EDTA by centrifuging at an RCF (Relative Centrifugal Force) of 2000xg at room temperature (20 °C) for 10 minutes. Patients’ ctDNA was isolated from up to 5 ml of plasma using the QIAmp circulating nucleic acids Kit(Qiagen). The DNA concentration was determined and sequencing libraries generated using Inivata® eTAM-SeqTM assay designed to cover 34 regions from 34 cancer-related genes. The libraries were sequenced on InVisionSeq™ - Lung amplicon-based NGS.

### Sample size and statistical analysis

Using an A’Hern single stage design, we required 37 patients to detect a 6-month PFS rate of 30% (minimum of 15%), with one-sided 10% statistical significance and 80% power; at least 9 of 37 patients need to be alive and progression-free at 6 months. PFS, OS and time on treatment were analysed using Kaplan-Meier methods. Analyses were performed for all patients, as well as separately for those with confirmed or suspected EGFR mutations. Additional analyses reallocated patients who were identified as EGFR mutant through ctDNA.

## Results

### Patient characteristics

Between March 2013 and August 2015 a total of 39 patients were recruited to the study across 13 sites in the UK. The baseline characteristics are described in Table 1. The median age was 72 (range 36–90) and 77% of patients were female. 21 (54%) were confirmed tissue EGFR mutant (either at the time of registration or shortly after using diagnostic tissue), the most common mutations being L858R (9/21) and exon 19 deletion (7/21). Of the 18 patients with suspected (unconfirmed) EGFR mutations, 7 (39%) were former smokers, 10 (56%) had never smoked and 1 patient’s smoking status was unavailable.

**Table 1:** Baseline characteristics.

Characteristic	*N* = 39
**Age (years), median (range)**	72 (36–90)
**Age (years),** ***N*(%)**	
Under 70	18 (46.2)
70+	21 (53.8)
**Sex,** ***N*(%)**	
Female	30 (76.9)
Male	9 (23.1)
**WHO performance status,** ***N*(%)**	
0	6 (15.4)
1	21 (53.8)
2	11 (28.2)
3	1 (2.6)
**Stage,** ***N*(%)**	
IIIA	1 (2.6)
IIIB	7 (17.9)
IV	31 (79.5)
**EGFR genotype (either at registration or shortly after using diagnostic tissue),** ***N*(%)**	
Confirmed EGFR	21 (53.8)
*L858R*	*9 (23.1)*
*Exon 19 deletion*	*7 (17.9)*
*T790M (Exon 20)*	*1 (2.6)*
p. 767 A/ASVD insertion (*Exon 20)*	*1 (2.6)*
*G719S (Exon 18)*	*3 (7.7)*
Suspected EGFR	18 (46.2)
*Former Smoker*	*7 (17.9)*
*Never Smoked*	*10 (25.6)*
*Unknown (whether former/never)*	*1 (2.6)*
**EGFR mutation (using ctDNA)**	
Mutation detected	24 (61.5)
*L858R*	*8 (20.5)*
*Exon 19 deletion*	*9 (23.1)*
*Exon 19 deletion and T790M*	*1 (2.6)*
*p771 Insertion (Exon 20)*	*1 (2.6)*
*p733-744 Insertion (Exon 20)*	*1 (2.6)*
*R776H (Exon 18)*	*1 (2.6)*
*G719A (Exon 18)*	*1 (2.6)*
*G719A (Exon 18) & V834L (Exon 21)*	*1 (2.6)*
*L861Q*	*1 (2.6)*
No mutation	14 (35.9)
N/A	1 (2.6)

### Treatment compliance and toxicity

Median time on treatment for the whole group of proven and suspected EGFR mutant NSCLC was 4.4 months (range 0.2 to 101), with 7 (18%) who had been on afatinib for ≥18 months, 3 for ≥36 months and 2 still ongoing as of June 2023 (101 months each). Disease progression was the most common cause for stopping treatment (44%), followed by unacceptable toxicity or serious adverse event (SAE) (31%). Around half of the patients received a reduced dose at some point during treatment (54%), mainly due to diarrhoea (12 patients). Two patients stopped trial treatment before restarting afatinib off trial shortly after (a maximum treatment break of 1 month was protocol-mandated). For analysis it was assumed that their treatment was continuous. 87% of patients experienced a grade ≥3 adverse event during trial treatment, the most common being diarrhoea (31%), vomiting (15%) and hypertension (15%) (Supplementary Table S1). There were 5 fatal events reported (2 pneumonitis, 1 acute kidney injury, 1 lung infection and 1 left ventricular failure). As expected, afatinib in this comorbid population was associated with a high number of treatment-related adverse events (97% all grade, 56% grade 3 + ) (Supplementary Table S2).

### Efficacy

We obtained long-term follow up (median 98 months, range for survivors 69 to 101). In the whole group, 28% (11/39) did not progress before 18 months, and 5 were progression-free beyond 36 months (3 L858R and 2 exon 19 deletion); of which only 3 have since progressed or died. 9 (23%) patients survived beyond 3 years (8 EGFR-mutant, 1 suspected) of which 4 were still alive at last follow up (at 69, 98, 101 and 101 months for each).

Median PFS and OS for all patients were 7.9 months (95% CI 4.6-10.5) and 15.5 months (95% CI 10.0-27.5), respectively. Despite a clear difference in PFS and OS between confirmed and (baseline) suspected EGFR mutant cases (median PFS months 10.5 confirmed vs 3.2 suspected; median OS months 29.7 confirmed vs 10.0 suspected), 6-month PFS rates far exceeded the 30% target for all patients as well as in the separate cohorts, with rates of 59% (95% CI 42-72) for all patients, 76% (52–89) for confirmed and 39% (17–60) for suspected (Table 2). The effect of afatinib was greatest for the 16 patients with exon 19 deletion or L858R, with median PFS of 13.0 months (95% CI 5.9-39.3) and median OS of 33.4 months (95% CI 13.8-54.5), while the 5 patients with other types of EGFR mutations had similar outcomes to those with only suspected mutations (Supplementary Fig. S2).

**Table 2:** Efficacy summary.

	PFS (36 events)		OS (35 deaths)	
	**All patients**			
	**(*N*** = **39)**			
6 months (95% CI)	59% (42–72)		74% (58–85)	
12 months	33% (19–48)		62% (45–75)	
36 months	13% (5–25)		23% (11–37)	
Median, months	7.9 (4.6–10.5)		15.5 (10.0–27.5)	
	**EGFR status at baseline (tissue)**			
	**Confirmed**	**Suspected**	**Confirmed**	**Suspected**
	**(*N*** = **21)**	**(*N*** = **18)**	**(*N*** = **21)**	**(*N*** = **18)**
6 months	76% (52–89)	39% (17–60)	86% (62–95)	61% (35–79)
12 months	48% (26–67)	17% (4–37)	81% (57–92)	39% (17–60)
36 months	24% (9–43)	0%	38% (18- 58)	6% (0–22)
Median, months	10.5 (6.5–27.5)	3.2 (2.6–7.9)	29.7 (13.8–47.7)	10.0 (3.9–16.3)
	**EGFR status at baseline (ctDNA)**			
	**EGFR mutant**	**Not EGFR mutant**	**EGFR mutant**	**Not EGFR mutant**
	**(*N*** = **24)**	**(*N*** = **14)**	**(*N*** = **24)**	**(*N*** = **14)**
6 months	58% (36–75)	64% (34–83)	75% (53–88)	79% (47–93)
12 months	38% (19–56)	29% (9–52)	67% (44–82)	57% (28–78)
36 months	13% (3–29)	14% (2–37)	25% (10–43)	21% (5–45)
Median, months	7.9 (4.6–14.3)	6.5 (2.6–23.7)	14.8 (6.7–33.4)	16.3 (4.1–34.9)
	**Exon 19 deletion or L858R (tissue or ctDNA)**^**a**^			
	**Yes**	**No**	**Yes**	**No**
	**(*N*** = **21)**	**(*N*** = **18)**	**(*N*** = **21)**	**(*N*** = **18)**
6 months	71% (47–86)	44% (22–65)	86% (62–95)	61% (35–79)
12 months	48% (26–67)	17% (4–37)	86% (62–95)	33% (14–55)
36 months	24% (9–43)	0%	33% (15–53)	11% (2–30)
Median, months	10.5 (5.9–19.8)	4.4 (2.6–6.7)	24.8 (14.2–39.3)	6.7 (4.0–16.3)
	**Baseline ctDNA values becoming undetectable during serial measurements**^**b**^			
	**ctDNA clearance**	**No clearance**	**ctDNA clearance**	**No clearance**
	**(*N*** = **14)**	**(*N*** = **4)**	**(*N*** = **14)**	**(*N*** = **4)**
6 months	86% (54–96)	0%	93% (59–99)	50% (6–84)
12 months	50% (23–72)	0%	86% (54–96)	50% (6–84)
36 months	21% (5–45)	0%	36% (13–59)	0%
Median, months	10.5 (6.7–19.8)	4.6 (2.8–N/A)	21.1 (12.6–54.5)	5.2 (4.8–N/A)

^a^Patients with rare mutations included within the “No” group had similar PFS/OS to those with no EGFR mutation identified (Fig. 2).

^b^Analysis excludes 6 patients without serial ctDNA and 15 without ctDNA detected EGFR mutations.

### ctDNA

**Fig. 1:**
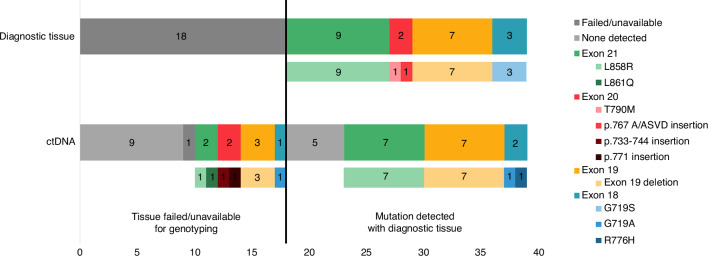
EGFR mutations. Tumour mutations detected using baseline tissue and ctDNA for all patients.

**Fig. 2:**
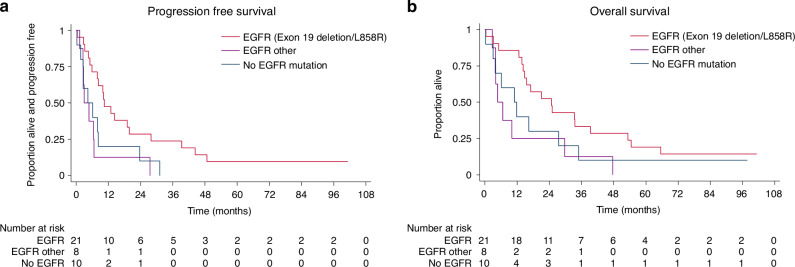
Survival outcomes by EGFR mutation type (tissue and ctDNA). **a** Progression-free survival and **b** Overall survival by EGFR mutation type, using both tissue and ctDNA.

Of the whole population (38 cases) tested by ctDNA, 79% (30 cases) had informative results (ctDNA identified). 8/18 (44%) of those cases suspected EGFR mutant at baseline (i.e. tissue failed/unavailable for genotyping) were subsequently identified by ctDNA to be EGFR mutant (Fig. 1). Of those, 4 had common EGFR mutations (exon 19 deletion or L858R) and 4 had rare mutations (Supplementary Table S4). Conversely, there were 5 patients with confirmed EGFR mutations from baseline tissue which were not identified on ctDNA, including 3 cases of tissue identified G719S, giving a false negative rate of 17% (5/29). Using EGFR by ctDNA, where a mutation was any type, there was very little difference in PFS and OS between those with and without known mutations (Supplementary Fig. 3). This is seemingly due to the poor prognosis or activity of afatinib in patients with rare EGFR mutations (excluding exon 20 insertions) only identified on ctDNA (Supplementary Fig. S4) as well as the failure of ctDNA to identify EGFR mutant cases confirmed by tissue (3/5 of which had exon 19 deletion or L858R). When combining the EGFR results from tissue and ctDNA, there are 29 patients in total who have any EGFR mutation type, of which 21 have an exon 19 deletion or L858R mutation (5 more cases than the 16 when using only the baseline tissue where available). These patients have particularly good prognoses (Fig. 2), with 6-month PFS of 71% and median OS of 25 months (Table 2).

**Fig. 3:**
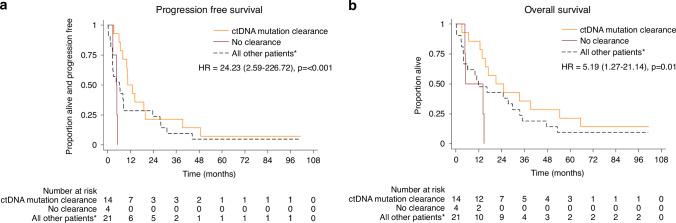
Survival outcomes by ctDNA mutation clearance. **a** Progression-free survival and **b** overall survival by whether baseline ctDNA became undetectable during serial measurements. *6 cases without serial ctDNA measurements and 15 without mutations.

Molecular complete response (defined as clearance of EGFR mutation detected through ctDNA with VAF < 0.03) was identified in 14/18 ctDNA-detected EGFR mutant patients with serial ctDNA samples (13 at 12 weeks, 1 at 24 weeks) and was a strong predictor of better OS (*p* = 0.01) and PFS (*p* < 0.001) compared to the 4 cases who did not clear circulating EGFR (Fig. 3). The 4 cases who did not clear EGFR had p.771:N/SVDN Insertion (exon 20), exon 19 deletion with T790M, exon 19 deletion alone, and exon 19 deletion with EGFR amplification. There was a strong correlation between RECIST response and ctDNA clearance, with 13/14 patients with ctDNA clearance achieving partial response (PR) and 1/14 stable disease (SD) compared to 1/4 PR and 3/4 SD without ctDNA clearance (Supplementary Table S3), and best response was indeed strongly associated with OS (*p* = 0.008) and PFS (*p* = 0.002). Interestingly, the patient with SD and ctDNA clearance had a long PFS time of 39.3 months, while the patient with PR and no clearance had a short time to progression of just 4.6 months. This implies that ctDNA clearance may be a better predictor of patient outcome than RECIST response, albeit numbers are far too small to confirm with any certainty.

**Fig. 4:**
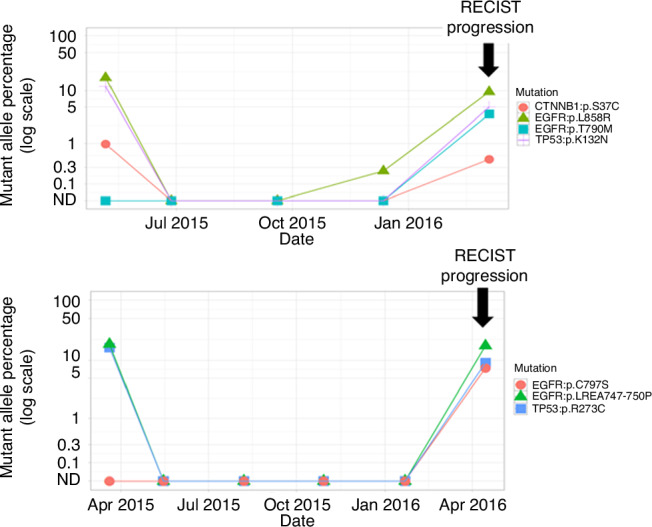
Examples of two patients with ctDNA monitoring for disease progression. ND Not Detectable.

In progressing patients with adequate sampling for analysis 6/10 patients with common EGFR mutations developed on-target resistance mutations, 2/10 developed off-target resistance and 1/10 had no change to their mutational profile and 1/10 cleared the EGFR mutation but retained the TP53 mutation. The median time from scan showing progressive disease to last ctDNA sample was 1 day (range −4 to 14). On-target acquired EGFR mutations were observed in 6/7 (86%) specifically, 6 exon 20 mutations were identified, each separately (T790M, C797S) (Supplementary Table S4). 5/6 cases with exon 19 deletion of EGFR had T790M (4/6) or C797S (1/6) on their last ctDNA sample. Three patients in total acquired off-target resistance mutations: 1 patient with EGFR L858R and TP53 at baseline acquired a CDK2NA resistance mutation, 1 patient with EGFR exon 19 and T790M at baseline acquired a TP53 mutation and 1 patient with suspected EGFR mutant disease (not verified) acquired an IDH mutation on progression. Figure 4 illustrates ctDNA monitoring for disease progression for two patients.

## Discussion

In this study, we examined previously untreated NSCLC patients considered clinically unsuitable for chemotherapy in two groups, confirmed and suspected EGFR mutant, to evaluate the efficacy and safety of afatinib in both groups and explore the impact of ctDNA analysis. Patients considered clinically unsuitable for chemotherapy would ordinarily have been managed by supportive care, only. The trial was conducted when neither osimertinib nor immune checkpoint inhibitors had been developed, when tissue NGS was not performed in England, and when ctDNA technology was experimental.

As expected, the proportion of patients with PS > 1 was higher in our population than those treated in real-world populations (31% TIMELY vs 7%–19% real-world [12–14]). Though most patients were PS 0–1, they had baseline comorbidities and were considered locally by investigators unfit to receive standard platinum therapy and would therefore have been ineligible for randomisation to the seminal LUX-Lung 3 and LUX-Lung 6 trials which each had a chemotherapy control arm [7, 8]. Reasons for chemotherapy contraindication were not captured. Given established failure rates and prevalence of inadequate specimens for genotyping, we did not exclude patients with suspected EGFR mutations (defined as never or ex-light smoking tobacco exposure) who would otherwise be treated with supportive care only, as other druggable oncogenic drivers had not been discovered at the time of trial initiation. Such patients were treated with afatinib on the basis of a proportion likely harbouring EGFR mutations as previously identified in the CALGB30406 trial of erlotinib in suspected EGFR mutant patients where a 40% EGFR mutation rate was identified in this same clinical demographic [19]. Indeed, our retrospective ctDNA testing of the suspected EGFR mutant group identified 44% of this group did in fact harbour EGFR mutations consistent with CALGB30406. Moreover, our ctDNA results confirm that ctDNA NGS is complimentary to tissue genotyping for drug target identification, being able to identify druggable genotypes in patients with inadequate tissue for genotyping, a practice that is now endorsed by clinical guidelines [20] and is routine in many parts of Asia where EGFR mutation prevalence rates are high, moving over to a “liquid-first” strategy [21]. Our finding that 5/21 (24%) of EGFR mutant patients based on tissue genotyping had non-informative ctDNA specimens at baseline is consistent with larger scale studies of tissue vs ctDNA NGS [18, 22]. Our data also identified a case of EGFR R667H in ctDNA but not in tissue. This genotype is sometimes observed with common EGFR mutations, but our recent publication suggests it may be pathogenic and germline in origin, segregating within a family [23]. However, we were unable to verify the somatic or germline status of this specific variant reported here, due to lack of source material for germline validation.

Our dataset also confirms the utility of ctDNA testing to oncogenic driver resistance mechanisms. Acquired exon 20 “on target” EGFR resistance mutations were identified in 86% of progressing cases, predominantly T790M in patients with EGFR exon 19 deletion and expected. Uniquely, however, we also identified a case of C797S-associated afatinib resistance, and that both mechanisms occur in isolation, not synchronously. The latter 797 codon is the binding site for afatinib and the contribution of C797X variants to afatinib resistance is poorly characterised given that large scale analyses of on-target afatinib resistance mechanisms have not been systematically performed in trials, and the model systems and osimertinib development has focused on T790M. Whilst afatinib usage for common EGFR mutations is waning, it remains used for patients with uncommon mutations and the contribution of afatinib-associated C797X variants remains a therapeutic target with the ongoing development of C797X sensitive 4^th^ generation EGFR inhibitors.

Our data also highlights the emerging utility of ctDNA clearance as a pharmacodynamic early surrogate endpoint of PFS, supporting findings from other datasets [24–26]. Importantly, in the current time when treatment options for front line EGFR mutant NSCLC may soon range between EGFR TKI monotherapy, TKI-chemotherapy combination, or lazertinib-amivantamab, lack of ctDNA clearance may assist in patient selection for those in whom treatment intensification above TKI monotherapy may be most useful, and inversely, those in whom treatment deintensification to TKI monotherapy may suit best. Moreover, whilst limited by the poorer prognostic impact of those with informative ctDNA findings, our results are consistent with ctDNA clearance being used in drug development studies as an early efficacy endpoint, alongside duration of response.

Overall, our ctDNA analyses confirm that ctDNA use is complimentary to tissue genotyping for drug target identification, that clearance predicts improved TKI efficacy, that level tracks alongside disease efficacy status, and that acquired resistance, resistance mechanisms can be determined.

We also confirm that afatinib significantly improved PFS compared to historical controls and is an effective and tolerable treatment option for EGFR mutant comorbid patients, a group that has been poorly evaluated with afatinib to date, given its higher adverse event profile than first-generation EGFR TKIs, and the commercial development focus on registration trials in patients suitable to be randomised to chemotherapy. Our data demonstrate modest efficacy for patients with uncommon EGFR mutations and for whom novel next generation EGFR kinase inhibitors hold the most promise [27].

Few studies of EGFR TKI therapies have reported long-term efficacy data. Observations from the LUX-Lung 3, LUX-Lung 6 and LUX-Lung 7 randomised trials found that a small proportion of patients ( ~ 10%) treated with afatinib reported enduring responses and remained on treatment for ≥3 years [28]. It is encouraging that a similar extent of long-term benefit was seen in our population of comorbid patients where, with extended follow-up, we identified a subset of patients with sustained benefit; with 3 remaining on treatment and 5 remaining progression-free for over 3 years. Further work is needed to determine predictive characteristics of these long-term responders to help guide treatment choice, and better predict acquired resistance mechanisms.

We observed more grade 3+ adverse events than in previous phase 3 studies; for example our grade 3+ diarrhoea rate of 31% was much higher than the 5–14% reported with LUX-Lung 3 and 6 trials [7, 8]. It was also higher than a 12.5% rate identified in a Japanese phase 2 trial of afatinib in patients aged 70 and above but without comorbidities [10]. However, this is likely expected in our unfit and comorbid population, and rates of treatment discontinuation due to toxicity were acceptable. Osimertinib is likely to be an alternative for patients in the modern era with single arm trials of poor performance status patients or elderly patients demonstrating lower rates of grade 3+ diarrhoea [4–6].

In conclusion, afatinib is a viable treatment option for tissue or ctDNA-detected EGFR mutant NSCLC patients considered unsuitable for chemotherapy because of comorbidities, with a proportion of patients achieving long-term clinical benefit, although osimertinib may be an alternative option in the modern era. Analysis of plasma ctDNA in patients with unsuitable tissue increased the yield of EGFR mutant patients identified, thereby improving diagnostic capabilities and was shown to be an effective tool for ongoing disease monitoring, supporting its expanded use in this setting.

## Supplementary information

Supplementary material

## Data Availability

Requests for data sharing with a research proposal should be addressed to the corresponding author. Depending on the specific research proposal, the Sponsor will determine when, for how long, for which specific purposes, and under which conditions the requested data can be made available, subject to ethical consent.
