# Supplementary material for: Long term efficacy of first-line afatinib and the clinical utility of ctDNA monitoring in patients with suspected or confirmed EGFR mutant non-small cell lung cancer who were unsuitable for chemotherapy
Source: Br J Cancer. 2024 Dec 5;132(3):245–52. doi: 10.1038/s41416-024-02901-6 (PMC11790930; doi:10.1038/s41416-024-02901-6)
Supplement: Supplementary file 1 — Supplementary material [file 41416_2024_2901_MOESM1_ESM.docx]

***Long term efficacy of first-line afatinib and the clinical utility of ctDNA monitoring in patients with suspected or confirmed EGFR mutant non-small cell lung cancer who were unsuitable for chemotherapy***

**SUPPLEMENTARY MATERIAL**

**
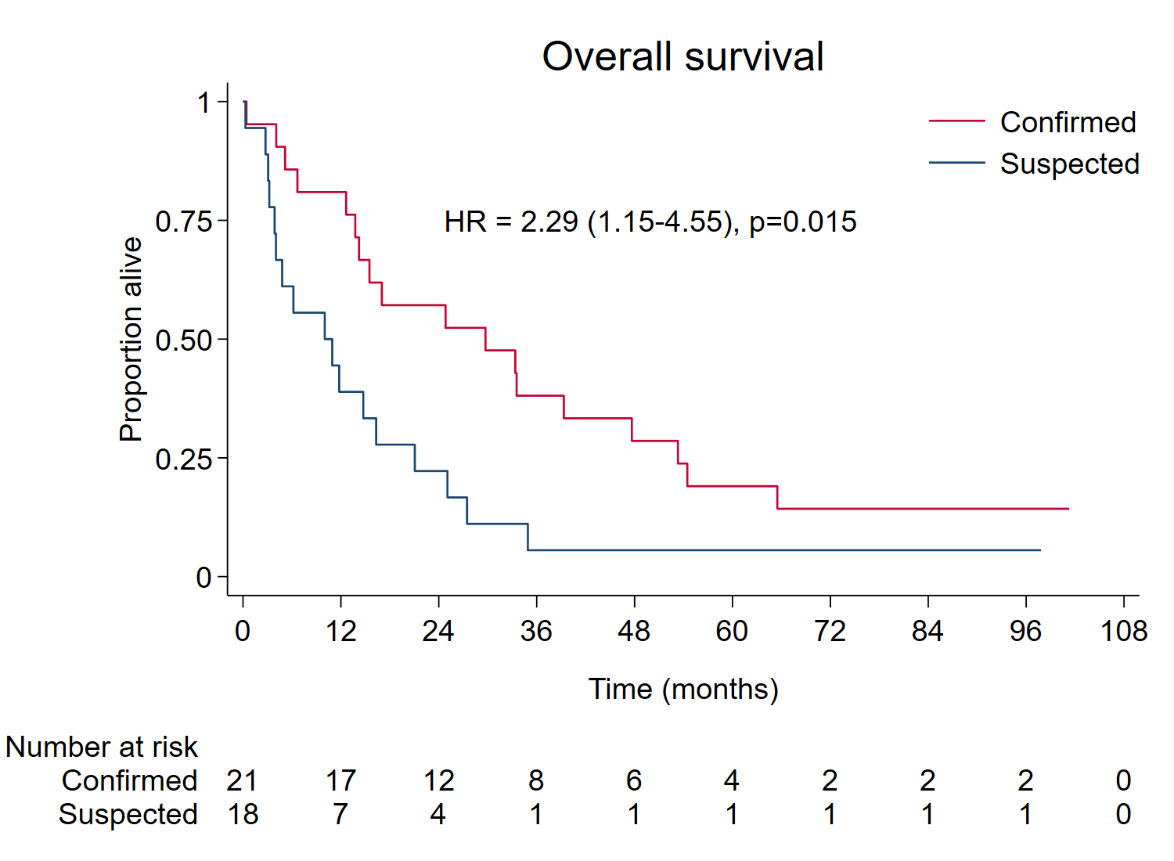

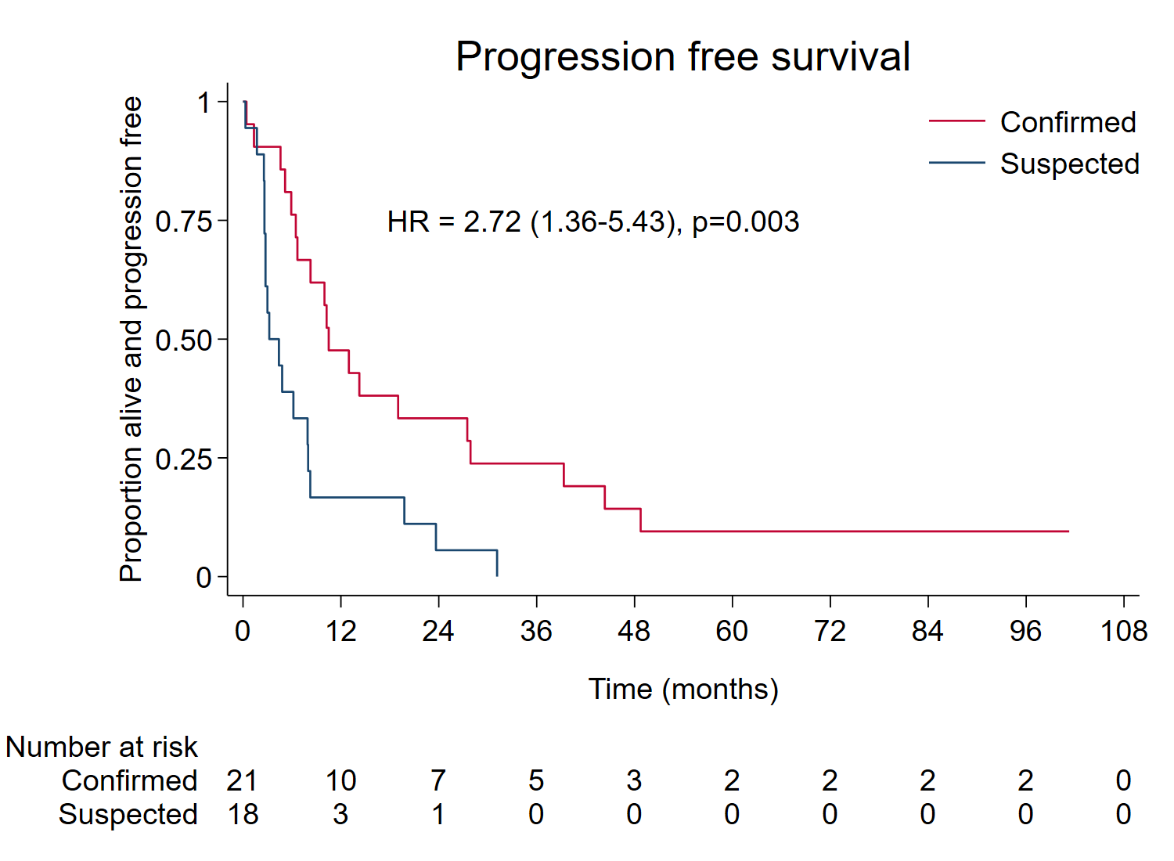

Figure S1: Using EGFR status at registration (tissue) (confirmed EGFR is any mutation)**

**
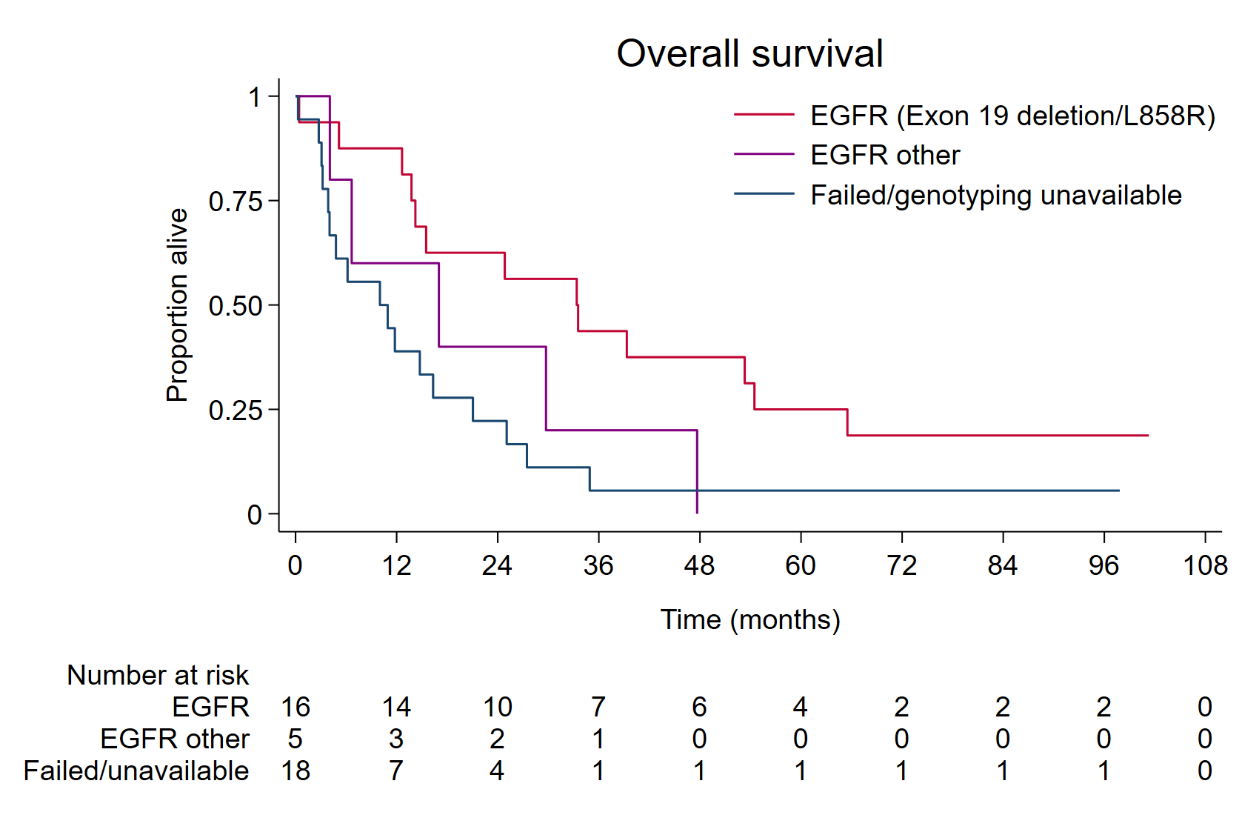

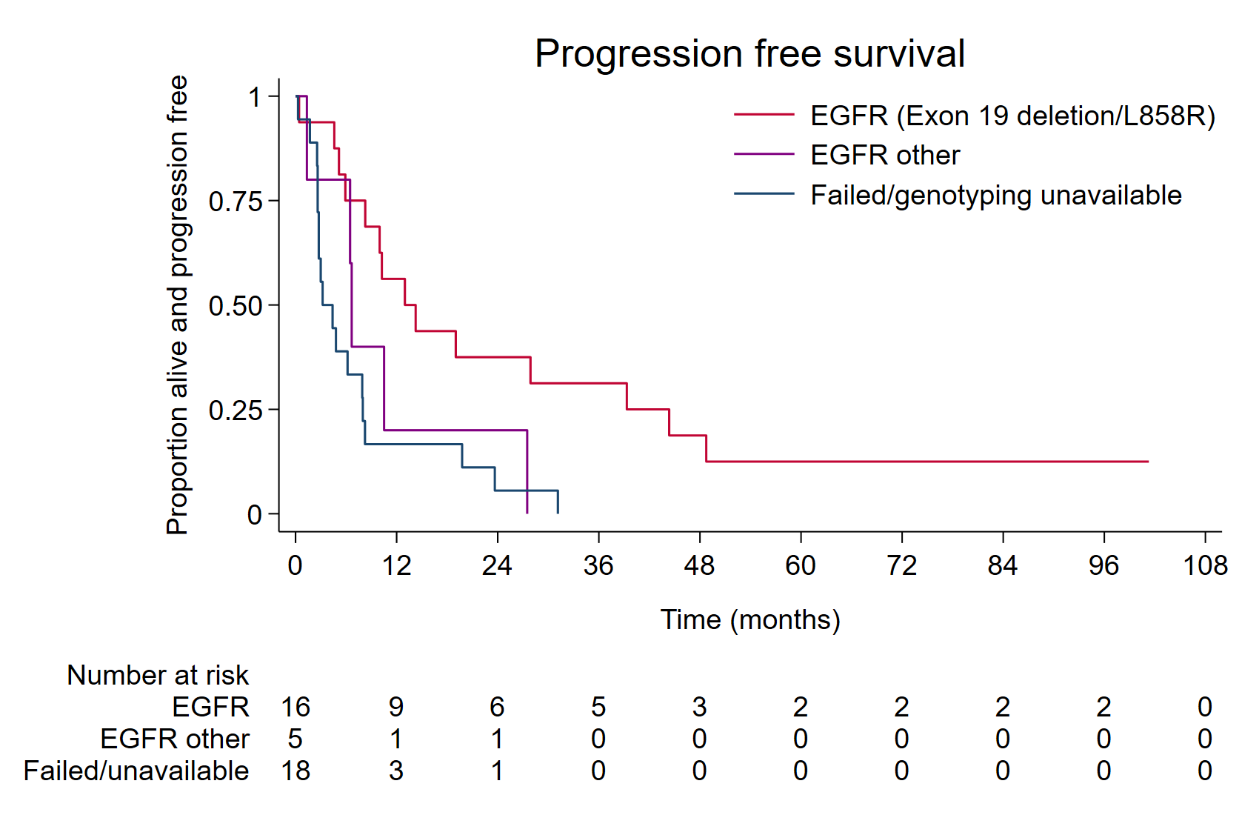
**
**Figure S2: Using EGFR status at registration (tissue), according to mutation type**

| "Other" mutations: |
| --- |
| P-GLY 719 ALA (x2) |
| T790M |
| Exon 20 deletion |
| Exon 18 deletion |
| Plen 747 & 751 deletion |

**
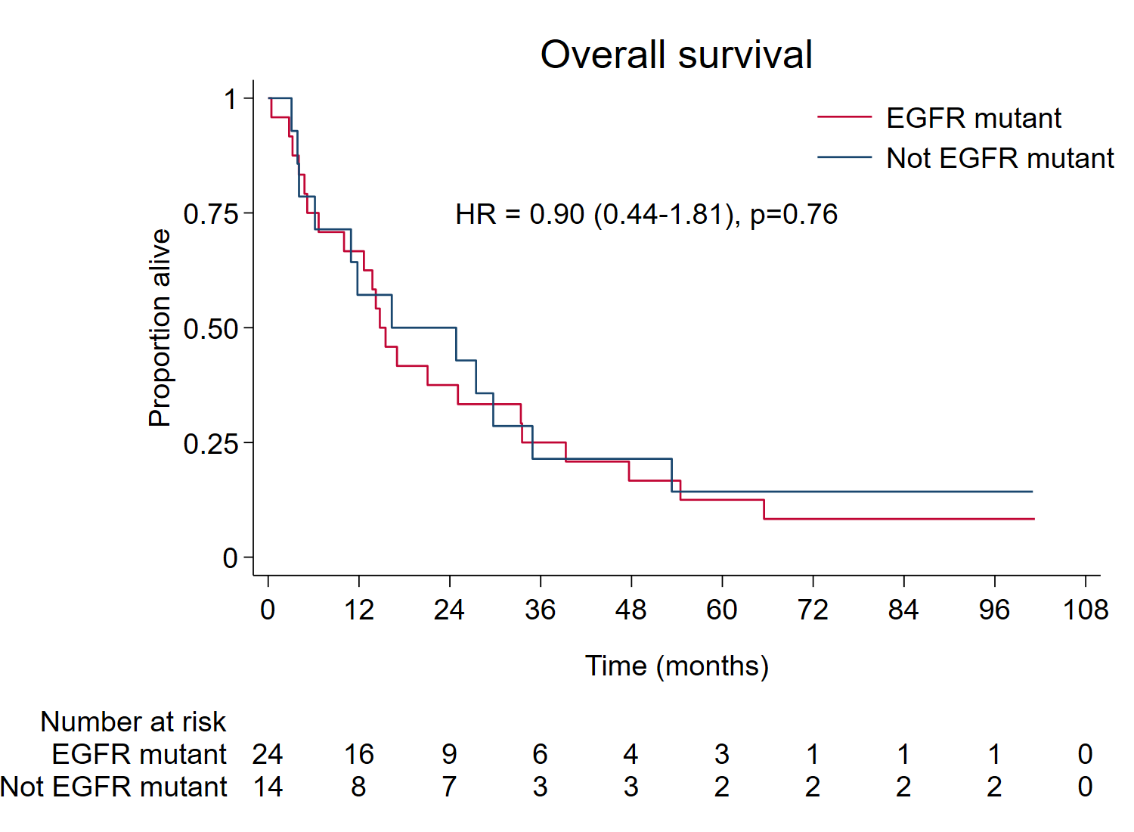

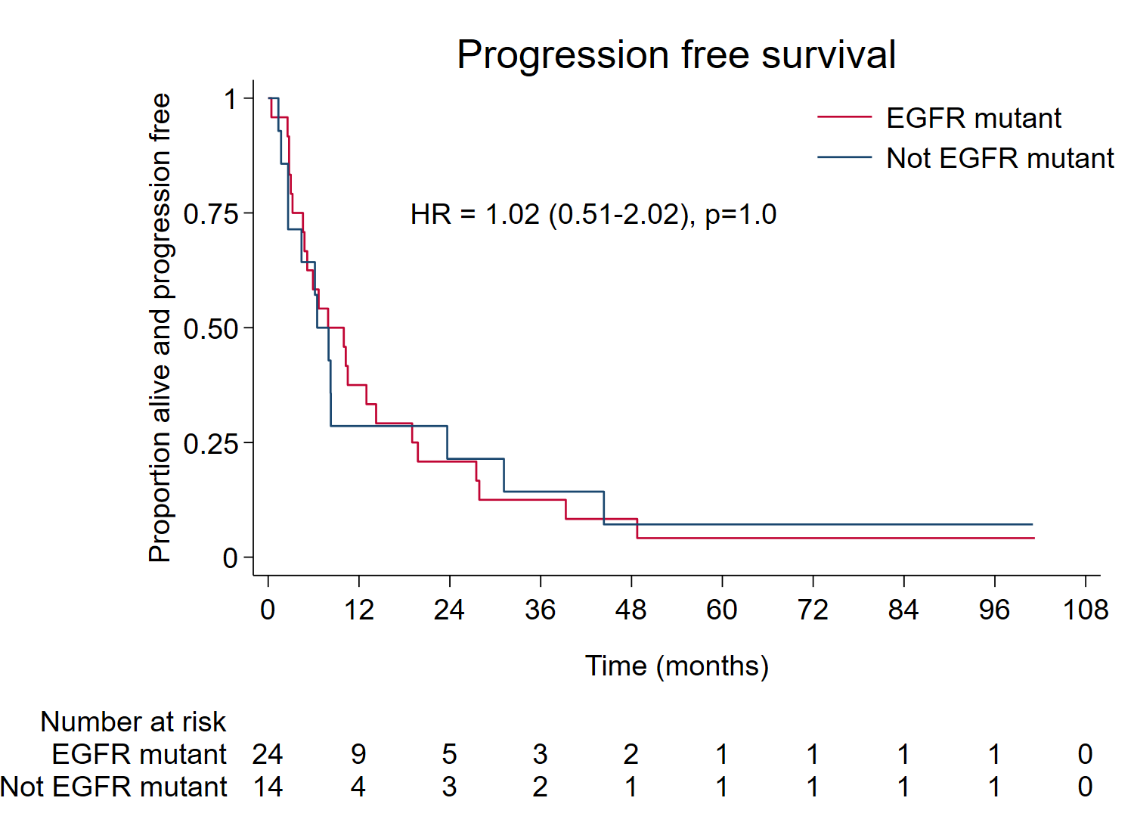
Figure S3: Using EGFR status by ctDNA (confirmed EGFR is any mutation)**


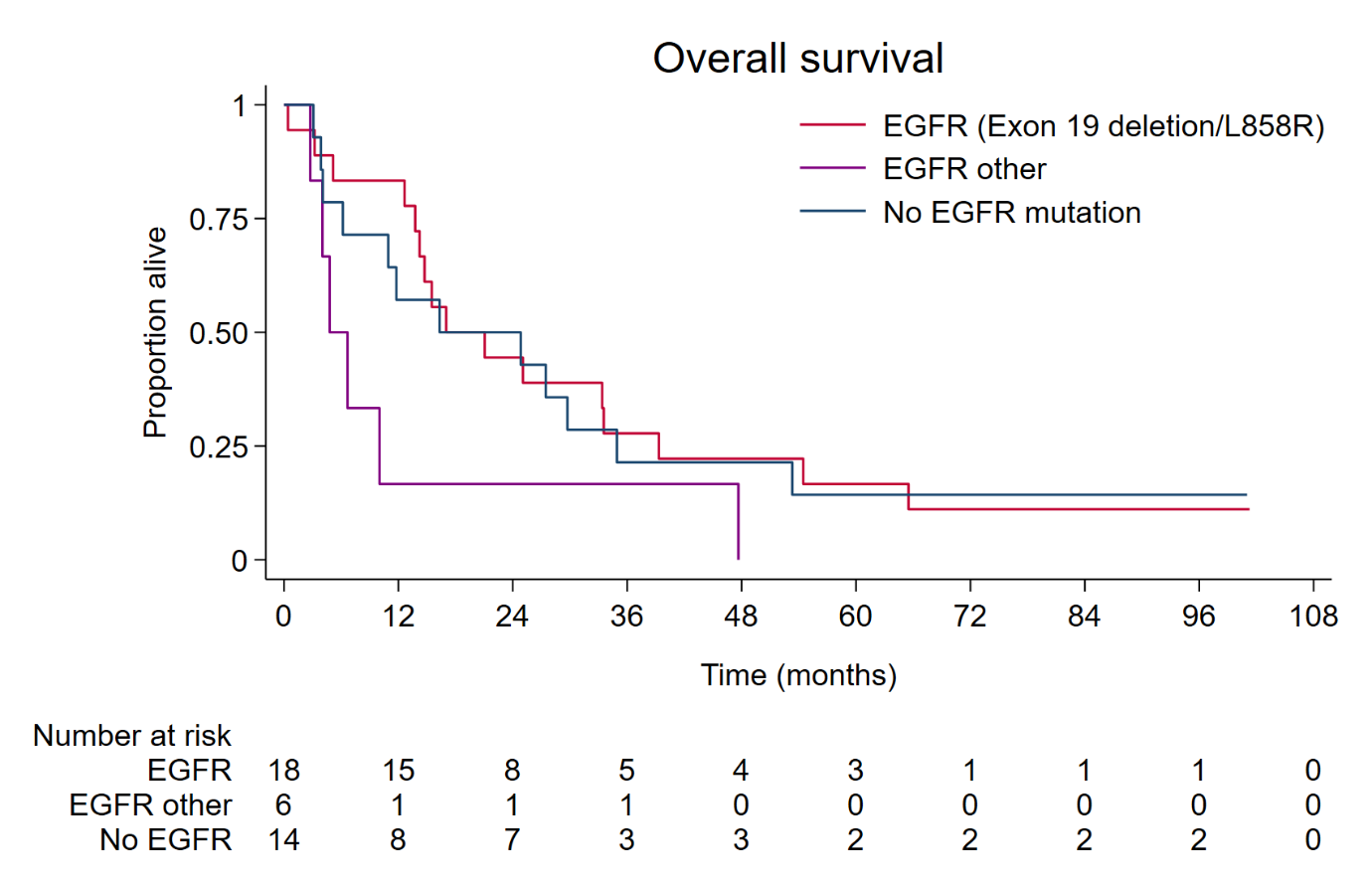

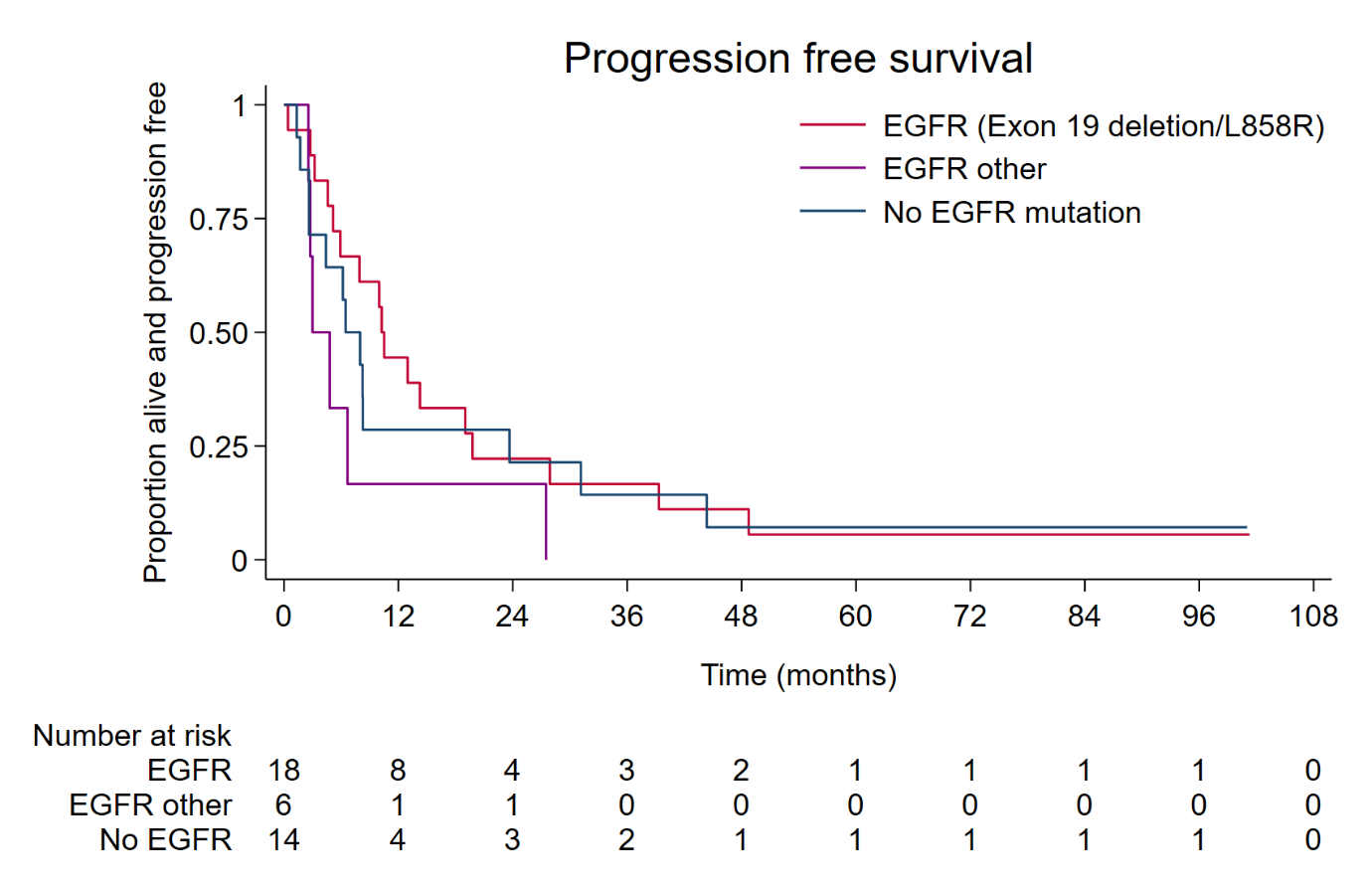


**Figure S4: Using EGFR status by ctDNA, according to mutation type (excluding Exon 20 insertions)**

| Other mutations: |  |
| --- | --- |
| G719A (Exon 18) & G598V (exon 14(15)) |  |
| G719A (exon 18) & V834L (exon 21) |  |
| L861Q |  |
| R776H (Exon 18) |  |

**Table S1: All observed toxicities of grade 3 or above**

| **Adverse event** | | **Grade 3** | **Grade 4** | **Grade 5** |
| --- | --- | --- | --- | --- |
|  |  | **N=39** | | |
| **Blood and lymphatic system disorders** | | **2 (5.1)** | **0** | **0** |
|  | Anemia | 2 (5.1) | 0 | 0 |
| **Cardiac disorders** | | **0** | **0** | **1 (2.6)** |
|  | Other: Left ventricular failure | 0 | 0 | 1 (2.6) |
| **Gastrointestinal disorders** | | **16 (41.0)** | **0** | **0** |
|  | Diarrhoea | 12 (30.8) | 0 | 0 |
|  | Nausea | 4 (10.3) | 0 | 0 |
|  | Other: Mouth ulcers | 1 (2.6) | 0 | 0 |
|  | Vomiting | 6 (15.4) | 0 | 0 |
| **General disorders and administration site conditions** | | **5 (12.8)** | **0** | **0** |
|  | Fatigue | 3 (7.7) | 0 | 0 |
|  | Pain | 1 (2.6) | 0 | 0 |
| **Infections and infestations** | | **6 (15.4)** | **2 (5.1)** | **1 (2.6)** |
|  | Bronchial infection | 0 | 1 (2.6) | 0 |
|  | Lung infection | 2 (5.1) | 0 | 1 (2.6) |
|  | Nail infection | 1 (2.6) | 0 | 0 |
|  | Other: Chest infection | 1 (2.6) | 0 | 0 |
|  | Other: E. Coli infection in urine | 1 (2.6) | 0 | 0 |
|  | Other: Lower respiratory infection | 1 (2.6) | 0 | 0 |
|  | Other: Norovirus | 1 (2.6) | 0 | 0 |
|  | Paronychia | 2 (5.1) | 0 | 0 |
|  | Sepsis | 0 | 1 (2.6) | 0 |
| **Injury, poisoning and procedural complications** | | **1 (2.6)** | **0** | **0** |
|  | Other: Food poisoning | 1 (2.6) | 0 | 0 |
| **Investigations** | | **2 (5.1)** | **0** | **0** |
|  | Creatinine increased | 1 (2.6) | 0 | 0 |
|  | Neutrophil count decreased | 1 (2.6) | 0 | 0 |
| **Metabolism and nutrition disorders** | | **7 (17.9)** | **1 (2.6)** | **0** |
|  | Anorexia | 1 (2.6) | 0 | 0 |
|  | Dehydration | 3 (7.7) | 0 | 0 |
|  | Hypercalcemia | 1 (2.6) | 0 | 0 |
|  | Hypokalemia | 1 (2.6) | 1 (2.6) | 0 |
|  | Hypomagnesemia | 1 (2.6) | 0 | 0 |
|  | Hyponatremia | 2 (5.1) | 0 | 0 |
| **Musculoskeletal and connective tissue disorders** | | **1 (2.6)** | **0** | **0** |
|  | Other: Left loin pain | 1 (2.6) | 0 | 0 |
| **Renal and urinary disorders** | | **2 (5.1)** | **0** | **1 (2.6)** |
|  | Acute kidney injury | 2 (5.1) | 0 | 1 (2.6) |
| **Respiratory, thoracic and mediastinal disorders** | | **1 (2.6)** | **0** | **2 (5.1)** |
|  | Dyspnoea | 1 (2.6) | 0 | 0 |
|  | Pleural effusion | 1 (2.6) | 0 | 0 |
|  | Pneumonitis | 0 | 0 | 2 (5.1) |
| **Skin and subcutaneous tissue disorders** | | **1 (2.6)** | **0** | **0** |
|  | Dry skin | 1 (2.6) | 0 | 0 |
| **Surgical and medical procedures** | | **1 (2.6)** | **0** | **0** |
|  | Other: Toe nail surgery | 1 (2.6) | 0 | 0 |
| **Vascular disorders** | | **6 (15.4)** | **0** | **0** |
|  | Hypertension | 6 (15.4) | 0 | 0 |
| **Any** | | **27 (69.2)** | **2 (5.1)** | **5 (12.8)** |

**Table S2: All observed treatment-related toxicities**All adverse events that were considered possibly, probably or definitely related to trial treatment.

| **Adverse event** | | **Grade 1-2** | **Grade 3+** |
| --- | --- | --- | --- |
|  |  | **N=39** | |
|  |  |  |  |
| **Blood and lymphatic system disorders** | | **3 (7.7)** | **2 (5.1)** |
|  | Anemia | 1 (2.6) | 2 (5.1) |
|  | Other: Hypouricemia | 1 (2.6) | 0 |
|  | Other: Thrombocytosis | 1 (2.6) | 0 |
| **Cardiac disorders** | | **2 (5.1)** | **0** |
|  | Atrial fibrillation | 1 (2.6) | 0 |
|  | Heart failure | 1 (2.6) | 0 |
|  | Ventricular arrhythmia | 1 (2.6) | 0 |
| **Eye disorders** | | **11 (28.2)** | **0** |
|  | Dry eye | 2 (5.1) | 0 |
|  | Eye pain | 1 (2.6) | 0 |
|  | Keratitis | 3 (7.7) | 0 |
|  | Other: Blepharitis | 1 (2.6) | 0 |
|  | Other: Itchy eyes | 1 (2.6) | 0 |
|  | Other: Not specified | 1 (2.6) | 0 |
|  | Watering eyes | 3 (7.7) | 0 |
| **Gastrointestinal disorders** | | **23 (59.0)** | **15 (38.5)** |
|  | Abdominal pain | 4 (10.3) | 0 |
|  | Cheilitis | 6 (15.4) | 0 |
|  | Constipation | 1 (2.6) | 0 |
|  | Diarrhoea | 24 (61.5) | 11 (28.2) |
|  | Dry mouth | 5 (12.8) | 0 |
|  | Dyspepsia | 1 (2.6) | 0 |
|  | Mucositis oral | 32 (82.1) | 0 |
|  | Nausea | 16 (41.0) | 2 (5.1) |
|  | Oral pain | 1 (2.6) | 0 |
|  | Other: Hypersalivation | 1 (2.6) | 0 |
|  | Other: Mouth ulcers | 0 | 1 (2.6) |
|  | Other: Not specified | 1 (2.6) | 0 |
|  | Other: Stomatitis | 1 (2.6) | 0 |
|  | Other: Throat ulcers | 1 (2.6) | 0 |
|  | Vomiting | 11 (28.2) | 4 (10.3) |
| **General disorders and administration site conditions** | | **25 (64.1)** | **2 (5.1)** |
|  | Fatigue | 25 (64.1) | 2 (5.1) |
|  | Fever | 1 (2.6) | 0 |
|  | Other: Blood in stool | 1 (2.6) | 0 |
|  | Other: Dysphonia | 1 (2.6) | 0 |
|  | Pain | 4 (10.3) | 0 |
| **Infections and infestations** | | **18 (46.2)** | **4 (10.3)** |
|  | Conjunctivitis | 6 (15.4) | 0 |
|  | Folliculitis | 1 (2.6) | 0 |
|  | Lung infection | 1 (2.6) | 0 |
|  | Nail infection | 1 (2.6) | 0 |
|  | Other: Bacterial tonsilitis | 1 (2.6) | 0 |
|  | Other: Cellulitis | 1 (2.6) | 0 |
|  | Other: E. Coli infection in urine | 0 | 1 (2.6) |
|  | Other: Toe infection | 1 (2.6) | 0 |
|  | Otitis externa | 1 (2.6) | 0 |
|  | Paronychia | 13 (33.3) | 2 (5.1) |
|  | Sepsis | 0 | 1 (2.6) |
|  | Skin infection | 2 (5.1) | 0 |
|  | Thrush (oral) | 1 (2.6) | 0 |
|  | Urinary tract infection | 1 (2.6) | 0 |
| **Investigations** | | **13 (33.3)** | **2 (5.1)** |
|  | Alanine aminotransferase increased | 5 (12.8) | 0 |
|  | Alkaline phosphatase increased | 3 (7.7) | 0 |
|  | Aspartate aminotransferase increased | 4 (10.3) | 0 |
|  | Blood lactate dehydrogenase increased | 2 (5.1) | 0 |
|  | Creatinine increased | 6 (15.4) | 1 (2.6) |
|  | Hemoglobin increased | 1 (2.6) | 0 |
|  | Lymphocyte count decreased | 1 (2.6) | 0 |
|  | Neutrophil count decreased | 1 (2.6) | 1 (2.6) |
|  | Other: Elevated urea | 1 (2.6) | 0 |
|  | Weight loss | 2 (5.1) | 0 |
| **Metabolism and nutrition disorders** | | **15 (38.5)** | **5 (12.8)** |
|  | Anorexia | 8 (20.5) | 1 (2.6) |
|  | Dehydration | 0 | 3 (7.7) |
|  | Hyperglycemia | 1 (2.6) | 0 |
|  | Hyperkalemia | 1 (2.6) | 0 |
|  | Hypermagnesemia | 1 (2.6) | 0 |
|  | Hypoalbuminemia | 6 (15.4) | 0 |
|  | Hypokalemia | 3 (7.7) | 2 (5.1) |
|  | Hypomagnesemia | 3 (7.7) | 1 (2.6) |
|  | Hyponatremia | 1 (2.6) | 0 |
|  | Other: Hypersalivation | 1 (2.6) | 0 |
| **Musculoskeletal and connective tissue disorders** | | **3 (7.7)** | **0** |
|  | Back pain | 1 (2.6) | 0 |
|  | Other: Sore hands and feet | 1 (2.6) | 0 |
|  | Other: Swollen Calf | 1 (2.6) | 0 |
| **Nervous system disorders** | | **12 (30.8)** | **0** |
|  | Dizziness | 2 (5.1) | 0 |
|  | Dysgeusia | 5 (12.8) | 0 |
|  | Headache | 1 (2.6) | 0 |
|  | Lethargy | 1 (2.6) | 0 |
|  | Other: Neuropathy | 1 (2.6) | 0 |
|  | Paresthesia | 3 (7.7) | 0 |
|  | Peripheral sensory neuropathy | 1 (2.6) | 0 |
| **Psychiatric disorders** | | **1 (2.6)** | **0** |
|  | Insomnia | 1 (2.6) | 0 |
| **Renal and urinary disorders** | | **4 (10.3)** | **2 (5.1)** |
|  | Acute kidney injury | 0 | 2 (5.1) |
|  | Other: Decreased renal function | 1 (2.6) | 0 |
|  | Proteinuria | 3 (7.7) | 0 |
| **Respiratory, thoracic and mediastinal disorders** | | **17 (43.6)** | **2 (5.1)** |
|  | Cough | 2 (5.1) | 0 |
|  | Dyspnoea | 4 (10.3) | 0 |
|  | Epistaxis | 12 (30.8) | 0 |
|  | Other: Haemoptysis | 1 (2.6) | 0 |
|  | Other: Haemorrhage upper respiratory (nose) | 1 (2.6) | 0 |
|  | Other: Mucositis nose | 1 (2.6) | 0 |
|  | Pneumonitis | 0 | 2 (5.1) |
|  | Sore throat | 1 (2.6) | 0 |
| **Skin and subcutaneous tissue disorders** | | **33 (84.6)** | **0** |
|  | Alopecia | 10 (25.6) | 0 |
|  | Dry skin | 20 (51.3) | 0 |
|  | Erythema multiforme | 3 (7.7) | 0 |
|  | Hyperkeratosis | 1 (2.6) | 0 |
|  | Nail loss | 1 (2.6) | 0 |
|  | Nail ridging | 1 (2.6) | 0 |
|  | Other: Blisters on toes | 1 (2.6) | 0 |
|  | Other: Brittle nails | 2 (5.1) | 0 |
|  | Other: Cellulitis | 1 (2.6) | 0 |
|  | Other: Erythema | 1 (2.6) | 0 |
|  | Other: Erythema nodosum | 1 (2.6) | 0 |
|  | Other: Foot ulcer | 1 (2.6) | 0 |
|  | Other: Ingrowing toe nail | 1 (2.6) | 0 |
|  | Other: Lip ulcer | 1 (2.6) | 0 |
|  | Other: Nail cracks | 2 (5.1) | 0 |
|  | Other: Nail pulps | 1 (2.6) | 0 |
|  | Other: Not specified | 4 (10.3) | 0 |
|  | Other: Psoriatic rash | 1 (2.6) | 0 |
|  | Other: Rash | 2 (5.1) | 0 |
|  | Other: Sore and splitting fingertips | 1 (2.6) | 0 |
|  | Other: Sore itchy scalp | 1 (2.6) | 0 |
|  | Palmar-plantar erythrodysesthesia syndrome | 9 (23.1) | 0 |
|  | Pruritus | 6 (15.4) | 0 |
|  | Rash acneiform | 25 (64.1) | 0 |
|  | Rash maculo-papular | 9 (23.1) | 0 |
|  | Scalp pain | 1 (2.6) | 0 |
|  | Skin ulceration | 1 (2.6) | 0 |
| **Surgical and medical procedures** | | **1 (2.6)** | **0** |
|  | Other: Operative wound post in-grown toenail removal in local anaesthetic | 1 (2.6) | 0 |
| **Vascular disorders** | | **1 (2.6)** | **0** |
|  | Lymphedema | 1 (2.6) | 0 |
| **Any** | | **16 (41.0)** | **22 (56.4)** |

**Table S3: Best response to treatment**

| **Best response to treatment** | **All patients**  **(N=39)** | **EGFR status at baseline (tissue)** | | **Baseline ctDNA becoming undetectable during serial measurements** | |
| --- | --- | --- | --- | --- | --- |
|  |  | **Confirmed (N=21)** | **Suspected (N=18)** | **ctDNA clearance (N=14)** | **No clearance (N=4)** |
| Partial Response | 20 (51.3%) | 15 (71.4%) | 5 (27.8%) | 13 (92.9%) | 1 (25.0%) |
| Stable Disease | 13 (33.3%) | 3 (14.3%) | 10 (55.6%) | 1 (7.1%) | 3 (75.0%) |
| Stopped due to toxicity before any assessment | 2 (5.1%) | 1 (4.8%) | 1 (5.6%) | 0 | 0 |
| Stopped due to clinician/patient decision before any assessment | 2 (5.1%) | 1 (4.8%) | 1 (5.6%) | 0 | 0 |
| Died before assessment | 2 (5.1%) | 1 (4.8%) | 1 (5.6%) | 0 | 0 |

**Table S4: Line listings of patient EGFR mutation status and clearance**

| **EGFR mutation from baseline tissue** | **EGFR mutation from baseline ctDNA** | **Clearance of EGFR mutation detected through ctDNA** | **Timing of EGFR clearance** | **PFS months** | **Reason for stopping treatment** | **EGFR mutation from ctDNA at last assessment before PD on treatment** | **Days from PD scan to last ctDNA assessment** |
| --- | --- | --- | --- | --- | --- | --- | --- |
| Tissue failed/unavailable for genotyping | No EGFR mutation | N/A | N/A | 23.7 | Clinician decision | N/A | N/A |
| Tissue failed/unavailable for genotyping | No EGFR mutation | N/A | N/A | 1.7 | PD | N/A | N/A |
| Tissue failed/unavailable for genotyping | No EGFR mutation | N/A | N/A | 2.6 | PD | N/A | N/A |
| Tissue failed/unavailable for genotyping | No EGFR mutation | N/A | N/A | 8.2 | PD | N/A | N/A |
| Tissue failed/unavailable for genotyping | No EGFR mutation | N/A | N/A | 6.2 | Adverse event | N/A | N/A |
| Tissue failed/unavailable for genotyping | No EGFR mutation | N/A | N/A | 2.6 | Death | N/A | N/A |
| Tissue failed/unavailable for genotyping | No EGFR mutation | N/A | N/A | 8.0 | PD | N/A | N/A |
| Tissue failed/unavailable for genotyping | No EGFR mutation | N/A | N/A | 31.1 | Adverse event | N/A | N/A |
| Tissue failed/unavailable for genotyping | No EGFR mutation | N/A | N/A | 4.4 | PD | N/A | N/A |
| Tissue failed/unavailable for genotyping | Exon 19 deletion and EGFR amp | Yes | C3 | 7.9 | PD | Exon 19 deletion and T790M | 6 |
| Tissue failed/unavailable for genotyping | Exon 19 deletion | Yes | C3 | 19.8 | Adverse event | N/A | N/A |
| Tissue failed/unavailable for genotyping | Exon 19 deletion & T790M | No | N/A | 2.8 | PD | Exon 19 deletion and T790M | 1 |
| Tissue failed/unavailable for genotyping | L858R | Yes | C3 | 3.2 | Adverse event | N/A | N/A |
| Tissue failed/unavailable for genotyping | p733-744 Insertion (Exon 20) | N/A | N/A | 2.6 | PD | Only baseline sample available | N/A |
| Tissue failed/unavailable for genotyping | p771 Insertion (Exon 20) | No | N/A | 4.8 | Adverse event | N/A | N/A |
| Tissue failed/unavailable for genotyping | G719A (Exon 18) | N/A | N/A | 2.8 | Adverse event | N/A | N/A |
| Tissue failed/unavailable for genotyping | L861Q | N/A | N/A | 3.0 | Patient decision | N/A | N/A |
| Tissue failed/unavailable for genotyping | N/A | N/A | N/A | 0.3 | Adverse event | N/A | N/A |
| Tissue failed/unavailable for genotyping | No EGFR mutation | N/A | N/A | 101.0 (ongoing) | Ongoing | N/A | N/A |
| Exon 19 deletion | Exon 19 deletion | N/A | N/A | 0.4 | Death | Only baseline sample available | N/A |
| Exon 19 deletion | Exon 19 deletion | Yes | C3 | 14.3 | PD | Exon 19 deletion and T790M | 14 |
| Exon 19 deletion | Exon 19 deletion | Yes | C3 | 5.9 | PD | Only baseline sample available | N/A |
| Exon 19 deletion | Exon 19 deletion | Yes | C3 | 13.0 | PD | Exon 19 deletion and C797S | 1 |
| Exon 19 deletion | Exon 19 deletion | Yes | C3 | 39.3 | Clinician decision | Only baseline sample available | N/A |
| Exon 19 deletion | Exon 19 deletion and EGFR amp | No | N/A | 4.6 | PD | Exon 19 deletion, T790M and EGFR amp | 0 |
| L858R | No EGFR mutation | N/A | N/A | 8.3 | PD | N/A | N/A |
| L858R | No EGFR mutation | N/A | N/A | 44.3 | Adverse event | N/A | N/A |
| L858R | L858R | N/A | N/A | 27.9 | PD | Only baseline sample available | N/A |
| L858R | L858R and S768I | Yes | C3 | 19.0 | Adverse event | N/A | N/A |
| L858R | L858R | Yes | C3 | 10.2 | Adverse event | N/A | N/A |
| L858R | L858R | Yes | C3 | 48.8 | Adverse event | N/A | N/A |
| L858R | L858R | Yes | C3 | 101.3 (ongoing) | Ongoing | N/A  Although cycle 27 has EGFR H773L | N/A |
| L858R | L858R | Yes | C3 | 10.0 | PD | L858R and T790M | 0 |
| L858R | L858R | No | N/A | 5.2 | Adverse event | N/A | N/A |
| G719S (exon 18) | No EGFR mutation | N/A | N/A | 1.3 | Adverse event | N/A | N/A |
| G719S (exon 18) | G719A (exon 18) & V834L (exon 21) | Yes | C3 | 6.7 | Adverse event | N/A | N/A |
| G719S (exon 18) | R776H (Exon 18) | N/A | N/A | 27.5 | Treatment non-compliance | N/A | N/A |
| p. 767 A/ASVD insertion (exon 20) | No EGFR mutation | N/A | N/A | 6.5 | PD | N/A | N/A |
| T790M (exon 20) | Exon 19 deletion | Yes | C6 | 10.5 | PD | EGFR exon 19 deletion | -4 |
